# Frequent Promoter Hypermethylation of the *APC* and *RASSF1A* Tumour Suppressors in Parathyroid Tumours

**DOI:** 10.1371/journal.pone.0009472

**Published:** 2010-03-01

**Authors:** C. Christofer Juhlin, Nimrod B. Kiss, Andrea Villablanca, Felix Haglund, Jörgen Nordenström, Anders Höög, Catharina Larsson

**Affiliations:** 1 Department of Molecular Medicine and Surgery, Center for Molecular Medicine, Karolinska Institutet, Karolinska University Hospital Solna, Stockholm, Sweden; 2 Department of Oncology-Pathology, Center for Molecular Medicine, Karolinska Institutet, Karolinska University Hospital Solna, Stockholm, Sweden; University of Hong Kong, Hong Kong

## Abstract

**Background:**

Parathyroid adenomas constitute the most common entity in primary hyperparathyroidism, and although recent advances have been made regarding the underlying genetic cause of these lesions, very little data on epigenetic alterations in this tumour type exists. In this study, we have determined the levels of promoter methylation regarding the four tumour suppressor genes *APC*, *RASSF1A*, *p16^INK4A^* and *RAR-β* in parathyroid adenomas. In addition, the levels of global methylation were assessed by analyzing *LINE-1* repeats.

**Methodology/Principal Findings:**

The sample collection consisted of 55 parathyroid tumours with known *HRPT2* and/or *MEN1* genotypes. Using Pyrosequencing analysis, we demonstrate *APC* promoter 1A and *RASSF1A* promoter hypermethylation in the majority of parathyroid tumours (71% and 98%, respectively). Using TaqMan qRT-PCR, all tumours analyzed displayed lower *RASSF1A* mRNA expression and higher levels of total *APC* mRNA than normal parathyroid, the latter of which was largely conferred by augmented *APC 1B* transcription levels. Hypermethylation of *p16^INK4A^* was demonstrated in a single adenoma, whereas *RAR-β* hypermethylation was not observed in any sample. Moreover, based on *LINE-1* analyses, parathyroid tumours exhibited global methylation levels within the range of non-neoplastic parathyroid tissues.

**Conclusions/Significance:**

The results demonstrate that *APC* and *RASSF1A* promoter hypermethylation are common events in parathyroid tumours. While *RASSF1A* mRNA levels were found downregulated in all tumours investigated, *APC* gene expression was retained through *APC 1B* mRNA levels. These findings suggest the involvement of the Ras signaling pathway in parathyroid tumorigenesis. Additionally, in contrast to most other human cancers, parathyroid tumours were not characterized by global hypomethylation, as parathyroid tumours exhibited *LINE-1* methylation levels similar to that of normal parathyroid tissues.

## Introduction

Primary hyperparathyroidism (PHPT) is a common endocrine disorder which denotes the tumorous enlargement of one or more of the parathyroid glands. While the genetic background of PHPT has been partly elucidated, the involvement of epigenetic modifications remains unresolved. Mutations in the *HRPT2* (also known as *CDC73*) and *MEN1* tumour suppressor genes are two important genetic events propelling parathyroid tumorigenesis [1]–[3]. The protein product of *HRPT2* is parafibromin, which is functionally linked to the canonical part of the Wingless type (Wnt) pathway [4]. Parafibromin is a part of the human polymerase associated factor complex (hPAF) which is required for facilitating transcription elongation and histone modifications [5]–[6]. Furthermore, parafibromin has been shown to associate with the Wnt oncoprotein β-catenin and to down-regulate c-Myc oncogene transcription through direct binding to the c-Myc promoter region, supporting the role of parafibromin as a tumor suppressor protein that inhibits Wnt signaling [7]. Interestingly, the *MEN1* product menin was also coupled to the Wnt pathway, demonstrated by its epigenetic regulation of Axin2 and by expression profiling of *MEN1* knock-out mice [8]–[9].

To broaden the knowledge of epigenetic events in parathyroid tumour development, we sought to determine the levels of promoter methylation of four tumour suppressor genes; *APC* (adenomatous polyposis coli), *p16^INK4A^*, *RASSF1A* (Ras association domain family protein 1) and *RAR-β* (Retinoic acid receptor-beta), all known to be widely hypermethylated in various human cancers as well as exhibiting potential as parathyroid tumour suppressor genes. *APC* mutations are responsible for the autosomal dominant familial adenomatous polyposis (FAP) syndrome [10]–[11], and are also found in sporadic colorectal cancer as well as in other tumour types, such as cancer of the thyroid and mammary glands [12]–[13]. Hypermethylation of the *APC* promoter 1A has been demonstrated in e.g. cancer of the colorectum, breast and lung [14]–[15]. Furthermore, loss of APC immunoreactivity has recently been implicated in parathyroid malignant tumours [16]. The tumour suppressor protein p16^ink4A^ regulates the G_1_ to S phase transition of the cell cycle through its inhibition of the interaction between Cdk 4/6 and cyclin D1. Parathyroid tumours often demonstrate over-expression of cyclin D1, thus making the *p16^ink4A^* gene an interesting candidate for promoter methylation assays since *p16^ink4A^* inactivation might produce parallel oncogenic effects analogous to cyclin D1 up-regulation [17]. *p16^ink4A^* gene mutations have been sought in parathyroid tumours due to the presence of 9p21-pter deletions in small subsets of parathyroid adenomas; however, no mutations have been demonstrated [18].

The *RASSF1A* gene localized to 3p21 is epigenetically inactivated with high regularity in human cancers as well as in endocrine tumours such as pheochromocytomas and follicular thyroid cancer [19]–[21]. Interestingly, RASSF1A has been shown to down-regulate cyclin D1 expression in addition to its role as a Ras-binding protein, hypothetically making the *RASSF1A* gene a potential candidate as a parathyroid tumour suppressor gene [22]. *RAR-β* is a member of the nuclear receptor superfamily with the main function to convey retinoid signaling into target gene transcription in the nucleus. The *RAR-β* promoter exhibit frequent hypermethylation in various tumours, as for instance in breast- and prostate cancer as well as in malignant mesothelioma [23]–[25]. Recently, it was shown that up-regulation of the oncogene *c-myc* increased the levels of *RAR-β* promoter methylation in a prostate cell line, suggesting that these events might be directly or indirectly coupled [26]. These finding are of particular interest as c-myc over-expression has been demonstrated in parathyroid tumours [27].

In addition to gene-specific hypermethylation, global hypomethylation is a common characteristic in human cancers [28]–[29]. *LINE-1* (long interspersed nuclear elements-1) retrotransposons denote replicating repetitive elements which constitute about 15% of the human genome. CpG sites within the *LINE-1* promoter region are normally heavily methylated to protect from retrotransposon activation, and *LINE-1* hypomethylation is a noted epigenetic event in human tumourigenesis. For example, *LINE-1* promoter hypomethylation is common in colon cancer, and the retrotransposal insertion of a *LINE-1* sequence has been directly shown to disrupt the *APC* gene in a case of colon cancer [29]–[30]. Furthermore, quantifying *LINE-1* promoter methylation densities have been shown to be a reliable substitute for global methylation assays [31].

Towards these ends, we have quantified promoter methylation of *APC*, *RASSF1A*, *p16^INK4A^*, *RAR-β* and *LINE-1* repeats in a series of parathyroid tumours characterized for *HRPT2* and *MEN1* genotypes.

## Results

### The APC and RASSF1A Promoters Are Hypermethylated in Parathyroid Tumours

**Figure 1:**
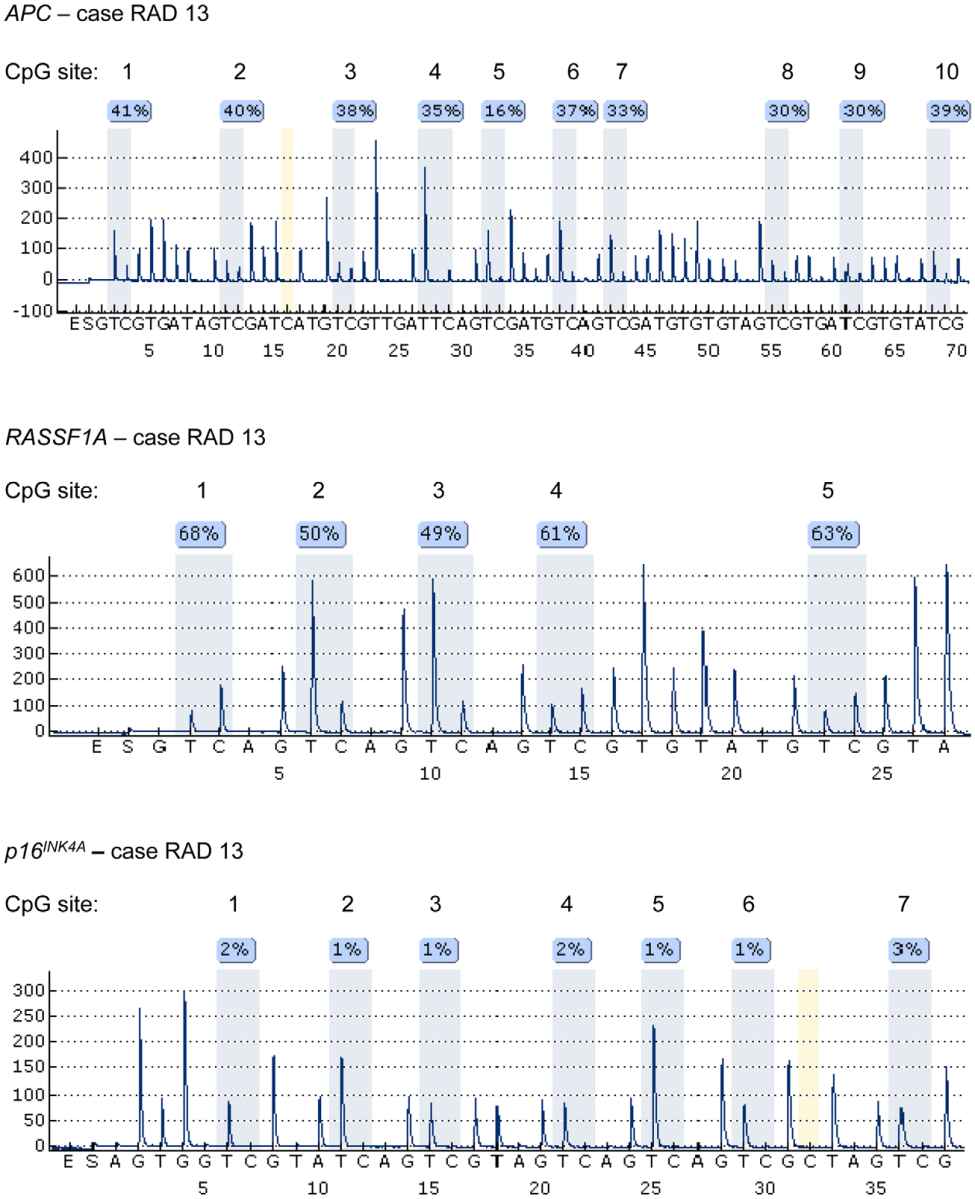
Examples of Pyrograms for quantification of methylation densities. 10 CpG sites of the *APC* promoter 1A, 5 CpG sites of the *RASSF1A* promoter and 7 CpG sites of the *p16^INK4A^* promoter are shown for case RAD 13, a tumour carrying a somatic *MEN1* mutation. The tumour displays methylation densities of 33.9% at *APC* (range 16–41%), 58.2% at *RASSF1A* (range 49–68%) and 1.6% at *p16^INK4A^* (range 1–3%), respectively. Nucleotides at position #16 of the *APC* promoter region 1A and at position #32 of the *p16^INK4A^* promoter (yellow) are examples of CpG free cytosine residues which serve as internal controls for satisfactory bisulphite-driven C to T conversion for each DNA sample.

**Figure 2:**
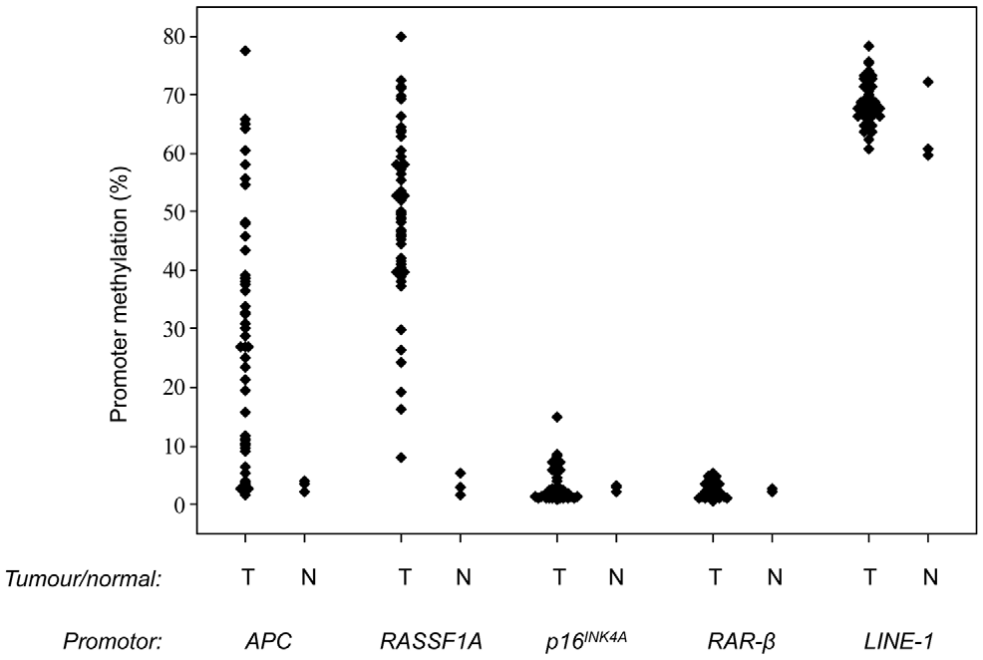
Global and regional hypermethylation in parathyroid tumours. Individual value plot illustrating the mean densities of CpG methylation of the *APC*, *RASSF1A*, *p16^INK4A^*, *RAR-β* and *LINE-1* promoters for the 55 parathyroid tumours (T) and 3 normal parathyroid controls (N). The Y axis represents the mean density of methylation calculated as an average of the individual CpG residues assayed.

In total, 55 parathyroid tumors were assessed for *APC*, *RASSF1A*, *p16^INK4A^* and *RAR-β* regional promoter methylation using Pyrosequencing analysis. Examples of Pyrograms are illustrated in Figure 1 and the results are summarized in Table 1. The density of promoter methylation in each tumour was calculated as a mean for the individual CpG residues, and the results are also presented as the range from minimum to maximum methylation density for the individual sites assessed (Table 1). The results indicate that CpG-specific methylation in parathyroid tumours is a common event at the *APC* and *RASSF1A* promoters but rarely occur at the *p16^INK4A^* and *RAR-β* promoter loci (Figure 2). For *APC*, the mean CpG methylation ranged from 1.5% (case RAD1) to 77.5% (case H2) in our panel (Table 1). Normal parathyroid tissues (Normal PT 1–3) displayed very low levels of *APC* promoter methylation (2.2%, 4.0% and 3.3%; Table 1). In addition, SHPT1, thymus tissue from case H1 as well as leukocyte DNA from patient RAD2 also exhibited low *APC* methylation (2.3% and 2.5%), in agreement with previous report of low *APC* methylation density in other non-neoplastic tissues such as colonic mucosa (≤5%) [32]. In our study, a total of 39 out of 55 tumours (71%) were considered to exhibit a hypermethylated *APC* promoter 1A (above 10% density) as compared to the 16 tumours (29%) with very low methylation (below 10% density). Twenty-four out of the 34 cystic parathyroid adenomas (71%) exhibited hypermethylation, as well as 14 out of 19 (74%) of the regular parathyroid adenomas. For the two cases with germ-line *HRPT2* gene mutations (H1-2), the atypical adenoma case H1 presented with virtually no methylation (2.3%), whereas the parathyroid adenoma H2 was endowed with the highest density of *APC* methylation in the entire series (77.5%).

**Table 1:** Results from the gene promoter methylation analyses in parathyroid tumours and control tissues.

Case	*APC* methylation †		*RASSF1A* methylation †		*p16^INK4A^* methylation †		*RAR-β* methylation †		*LINE-1* methylation †		Genotypes*	
no.	mean %	(range) %	mean %	(range) %	mean %	(range) %	mean %	(range) %	mean %	(range) %	*MEN1*	*HRPT2*
*Tumour samples*												
CAD1	2.7	(2–5)	39.6	(24–55)	8.1	(2–27)	1.0	(1–1)	66.3	(64–69)	wt	wt
CAD2	2.7	(2–4)	45.2	(34–59)	1.3	(1–2)	0.9	(0–2)	67.3	(66–70)	wt	wt
CAD3	2.9	(2–4)	46.8	(28–64)	2.1	(1–5)	1.1	(1–2)	67.0	(65–69)	wt	wt
CAD4	3.4	(2–4)	37.2	(22–48)	2.9	(1–5)	0.9	(0–1)	68.3	(65–71)	wt	wt
CAD5	3.6	(2–6)	50.0	(35–71)	4.4	(2–14)	1.0	(0–2)	67.7	(64–70)	wt	wt
CAD6	3.8	(3–5)	40.6	(27–57)	1.4	(1–2)	1.9	(1–3)	72.3	(70–75)	wt	wt
CAD7	3.9	(3–5)	49.0	(30–69)	1.9	(1–3)	1.7	(0–4)	64.7	(62–67)	wt	wt
CAD8	4.0	(3–5)	55.4	(41–71)	1.1	(1–2)	1.1	(1–2)	64.7	(62–66)	wt	wt
CAD9	5.3	(4–6)	38.0	(23–50)	0.9	(0–1)	0.8	(0–1)	64.0	(60–66)	wt	wt
CAD10	9.1	(6–13)	19.2	(9–31)	1.3	(1–2)	1.4	(0–3)	65.0	(64–67)	wt	wt
CAD11	10.3	(5–13)	48.8	(25–70)	1.6	(1–3)	1.6	(1–3)	71.3	(69–73)	wt	Mut (S)
CAD12	10.8	(7–26)	59.4	(48–67)	1.3	(1–2)	2.1	(1–4)	63.7	(61–66)	wt	wt
CAD13	15.8	(11–19)	8.0	(7–11)	5.4	(2–14)	0.4	(0–2)	72.7	(71–75)	wt	wt
CAD14	19.5	(12–26)	46.6	(32–59)	1.4	(1–2)	4.0	(2–8)	72.7	(70–77)	wt	wt
CAD15	23.4	(15–30)	52.8	(38–67)	2.6	(2–3)	1.4	(1–2)	68.0	(66–72)	wt	wt
CAD16	26.8	(8–38)	60.4	(49–73)	1.6	(1–3)	5.3	(3–7)	66.7	(63–69)	wt	wt
CAD17	26.9	(9–36)	39.6	(15–77)	2.7	(2–3)	1.3	(1–2)	65.3	(64–66)	wt	wt
CAD18	28.7	(11–37)	57.4	(47–67)	0.9	(0–2)	0.6	(0–3)	62.3	(60–64)	wt	wt
CAD19	30.0	(11–38)	50.2	(36–71)	1.0	(1–1)	1.0	(0–2)	70.7	(69–74)	wt	wt
CAD20	30.9	(16–36)	58.2	(30–86)	2.3	(1–4)	1.0	(1–1)	64.3	(61–67)	wt	wt
CAD21	31.0	(18–38)	24.2	(14–37)	3.9	(2–7)	1.9	(1–2)	73.3	(71–75)	wt	wt
CAD22	32.5	(16–42)	49.6	(36–59)	1.3	(1–2)	1.3	(1–2)	66.0	(64–68)	wt	wt
CAD23	32.8	(20–38)	49.8	(29–61)	2.4	(1–5)	2.9	(2–5)	66.7	(64–68)	wt	wt
CAD24	36.6	(16–45)	63.8	(60–69)	1.1	(1–2)	1.0	(1–1)	68.3	(66–72)	Mut (S)	wt
CAD25	37.6	(13–43)	69.4	(54–78)	1.3	(1–2)	2.4	(1–4)	69.7	(67–72)	wt	Mut (S)
CAD26	38.0	(26–57)	52.0	(29–61)	6.9	(4–12)	4.6	(3–8)	69.0	(67–70)	wt	wt
CAD27	39.2	(32–48)	44.4	(29–57)	1.1	(1–2)	2.1	(0–4)	64.6	(62–66)	wt	wt
CAD28	45.7	(18–69)	57.8	(27–77)	1.4	(1–3)	3.2	(2–7)	73.7	(72–77)	wt	wt
CAD29	48.0	(26–60)	29.8	(21–36)	0.9	(0–2)	1.6	(0–3)	66.3	(63–68)	wt	wt
CAD30	48.2	(38–55)	38.8	(21–49)	1.0	(1–1)	1.3	(1–2)	68.7	(65–71)	wt	wt
CAD31	54.5	(30–68)	45.8	(22–65)	2.7	(1–7)	1.1	(0–2)	66.3	(62–69)	wt	wt
CAD32	55.7	(44–63)	71.2	(64–78)	1.1	(1–2)	1.3	(0–4)	60.7	(58–63)	wt	wt
CAD33	60.6	(34–89)	45.4	(27–63)	8.6	(2–35)	1.6	(1–2)	68.7	(65–71)	wt	wt
CAD34	64.1	(39–78)	41.0	(25–50)	1.3	(1–2)	2.3	(1–4)	73.3	(70–75)	wt	Mut (S)
RAD1	1.5	(1–2)	71.4	(60–83)	2.3	(1–4)	2.9	(0–4)	75.3	(73–79)	wt	n.d.
RAD2	2.0	(1–4)	64.0	(55–72)	14.9	(2–34)	3.4	(0–7)	71.3	(70–72)	wt	n.d.
RAD3	2.5	(2–3)	66.4	(45–83)	5.9	(1–20)	2.7	(2–4)	67.3	(66–69)	wt	n.d.
RAD4	6.4	(2–23)	80.0	(63–96)	1.9	(1–4)	1.8	(0–2)	66.3	(65–67)	wt	n.d.
RAD5	9.6	(5–12)	48.2	(35–58)	7.1	(3–16)	3.2	(2–5)	67.3	(65–69)	wt	n.d.
RAD6	10.1	(5–27)	46.0	(29–70)	7.1	(3–15)	4.8	(3–7)	74.0	(72–76)	wt	n.d.
RAD7	11.1	(6–15)	42.2	(23–55)	1.1	(1–2)	3.4	(0–6)	68.3	(66–70)	wt	n.d.
RAD8	11.6	(7–17)	40.2	(24–49)	2.1	(1–4)	2.1	(0–6)	69.3	(67–71)	Mut (S)	n.d.
RAD9	21.3	(8–34)	53.2	(22–78)	1.3	(1–3)	3.4	(2–5)	67.7	(65–69)	wt	n.d.
RAD10	24.9	(17–31)	26.4	(14–46)	5.9	(1–11)	1.5	(0–3)	73.0	(71–75)	wt	n.d.
RAD11	26.8	(10–47)	41.6	(32–50)	1.1	(1–2)	3.1	(1–6)	71.7	(70–73)	wt	n.d.
RAD12	32.6	(20–43)	69.8	(63–76)	2.3	(1–5)	1.4	(1–2)	67.7	(65–71)	wt	n.d.
RAD13	33.9	(16–41)	58.2	(49–68)	1.6	(1–3)	2.0	(1–3)	70.0	(67–72)	Mut (S)	n.d.
RAD14	36.4	(23–46)	53.6	(46–62)	2.0	(1–5)	2.0	(1–4)	68.0	(65–71)	Mut (S)	n.d.
RAD15	38.6	(23–47)	56.4	(48–63)	0.9	(0–1)	0.9	(0–2)	67.7	(65–69)	Mut (S)	n.d.
RAD16	43.3	(17–53)	53.4	(36–71)	1.0	(1–1)	1.9	(0–3)	69.0	(66–72)	wt	n.d.
RAD17	58.0	(46–68)	64.4	(56–78)	1.3	(1–2)	4.8	(0–7)	68.7	(66–71)	wt	n.d.
RAD18	65.1	(50–78)	72.4	(56–91)	0.7	(0–1)	1.5	(0–8)	67.0	(64–70)	Mut (S)	n.d.
RAD19	65.9	(55–73)	63.0	(42–74)	1.9	(1–2)	2.8	(0–5)	63.7	(62–66)	Mut (S)	n.d.
H1	2.3	(1–5)	16.2	(13–19)	1.4	(1–2)	4.1	(2–7)	78.3	(75–83)	n.d.	Mut (G)
H2	77.5	(30–89)	52.8	(32–62)	1.9	(1–3)	2.6	(1–4)	75.7	(73–78)	n.d.	Mut (G)
*SHPT*												
SHPT1	2.5	(2–3)	30.2	(16–54)	3.0	(1–5)	1.6	(0–4)	71.3	(69–76)	n.d.	n.d.
*Normal controls*												
Normal PT1	4.0	(2–7)	5.2	(4–6)	2.9	(1–5)	2.1	(1–4)	60.7	(56–68)	n.d.	n.d.
Normal PT2	2.2	(1–5)	1.6	(1–2)	3.1	(1–7)	2.2	(1–4)	59.7	(55–67)	n.d.	n.d.
Normal PT3	3.3	(2–4)	3.0	(3–3)	2.1	(1–3)	2.6	(2–4)	72.3	(68–77)	n.d.	n.d.
Leukocyte RAD2	2.3	(1–4)	2.8	(2–3)	1.0	(1–1)	3.5	(2–6)	78.0	(75–80)	n.d.	n.d.
Thymus H1	2.4	(1–5)	3.8	(3–5)	1.6	(1–3)	6.8	(5–9)	77.3	(75–80)	n.d.	n.d.

†  =  Mean CpG methylation density in % (X% CpG1+Y% CpG2)n/n, range within parenthesis.

n.d: not determined; wt  =  wild-type, Mut (S/G)  =  somatic/germline mutation.

* Genotypes according to previous publications [3], [43]–[44].

For *RASSF1A*, the methylation ranged from 8% (CAD13) to 80% (RAD4) and 54 out of 55 (98%) tumours displayed methylation densities above 10% (Table 1, Figure 2). The three normal parathyroid samples presented with almost no *RASSF1A* promoter methylation and additional non-tumorous controls consisting of normal thymus and leukocyte DNA also displayed low levels of *RASSF1A* methylation. Case SHPT1 however exhibited an average methylation density of 30.2% (Table 1, Figure 2). There was a significant but weak positive correlation between *APC* and *RASSF1A* methylation levels, suggesting that tumours with high levels of *APC* methylation density frequently demonstrate high *RASSF1A* methylation levels (Pearson's correlation 0.301, p = 0.022, Table 2).

**Table 2:** Correlations between hypermethylation levels and tumour characteristics.

Parameter	*RASSF1A*	*APC*
*RASSF1A*	-	0.301 (p = 0.022)
*APC*	0.301 (p = 0.022)	-
Gender	n.s.	n.s.
Age at diagnosis	n.s.	n.s.
S-Ca	−0.417 (p = 0.003)	n.s.
S-PTH	n.s.	n.s.
Tumour weight	n.s.	n.s.
*MEN1* mutation	n.s.	0.322 (p = 0.027)
*HRPT2* mutation	n.s.	n.s.

n.s: not significant.

Regarding *p16^INK4A^* promoter methylation, one case was found to be hypermethylated as compared to non-tumorous controls (RAD2, 14.9%). The remaining 54 cases (98%) displayed very low mean levels of promoter methylation densities (<10%) (Table 1). However, nine of these cases were found to exhibit methylation levels above 10% at one or more individual CpG sites, with preferentially involvement of CpG site 7. For *RAR-β*, all tumours and normal controls displayed low levels of promoter methylation (<10%) (Table 1).

### Assessment of Global Methylation in Parathyroid Tumours

Regarding *LINE-1* promoter methylation, normal parathyroid tissues ranged from 59.7% to 72.3%, thereby encompassing the vast majority (45/55; 82%) of all parathyroid tumours investigated.

### Comparison of Promoter Methylation Density with Clinical and Genetic Parameters

Possible correlations were evaluated between methylation density of the individual hypermethylated *APC* and *RASSF1A* promoters with clinical and genetic characteristics (Table 1, Table 2, Table 3 and Table S1). No significant correlations were observed between *APC* promoter hypermethylation and serum levels of calcium, PTH or glandular weight (Table 2). For *RASSF1A*, no correlation between methylation levels and serum calcium, PTH or glandular weight was demonstrated; however, increased levels of *RASSF1A* methylation correlated negatively with serum calcium (Table 2).

**Table 3:** Summary of the parathyroid tumour study group.

Parameter		
*Number of samples/patients*	55	
*Sex*		
female	33	
male	22	
*Age at diagnosis*		
median (range) years	62 (16–86)	
*Familial disease*		
sporadic	52	
HPT-JT	2	
FIHP	1	
*Histopathology*		
adenoma	53	
atypical adenoma	2	
*S-Ca*		
median (range) mmol/L	2.85 (2.60–4.00)	
*S-PTH*		
median (range) ng/L	121 (36–711)	
*Tumor size*		
median (range) gram	1.24 (0.20–11.18)	
*MEN1 sequence*		
mutated	7	
wild-type	46	
n.a.	2	
*HRPT2 sequence*		
mutated	5	
wild-type	31	
n.a.	19	

n.a: not available.

**Figure 3:**
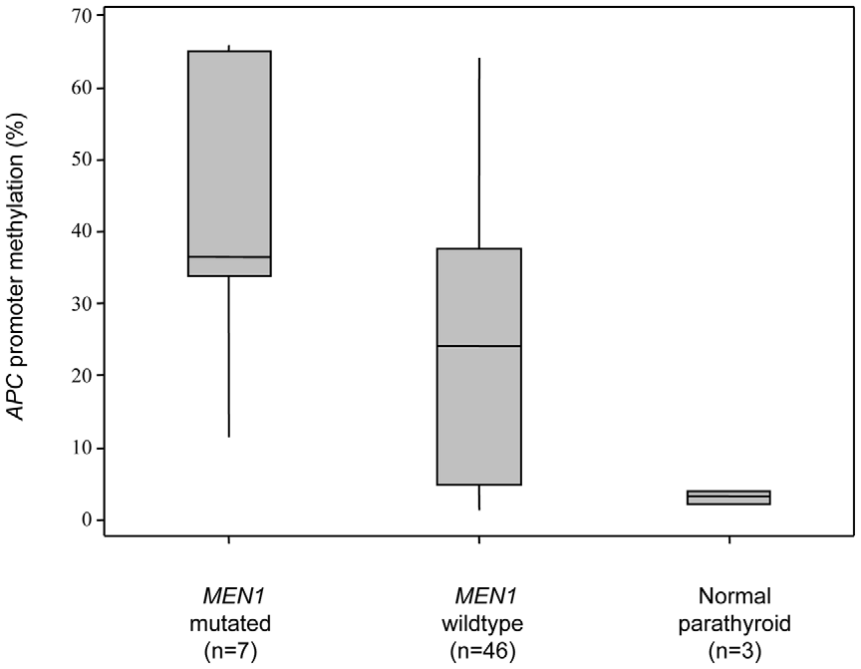
*APC* promoter 1A hypermethylation in parathyroid tumours with a coupling to *MEN1* mutations. Boxplot displaying *APC* promoter methylation in comparison to *MEN1* genotype for all cases where genotype data was available. The endpoints of the boxes represent the first and third quartiles respectively, and the horizontal line corresponds to the median. High *APC* promoter methylation density is statistically correlated to *MEN1* mutational status.

All *MEN1* mutated tumours in the current study displayed *APC* promoter 1A hypermethylation, and a weak but significant correlation (Pearson's correlation 0.322, p = 0.027) was revealed between high *APC* methylation density and presence of an *MEN1* mutation (Figure 3). However, no association was observed between *RASSF1A* methylation levels and *MEN1* genotype (Table 2). Furthermore, there was no correlation between *APC* or *RASSF1A* promoter methylation densities and *HRPT2* genotype (Table 2).

### APC and RASSF1A mRNA Expression Analyses by qRT-PCR

**Figure 4:**
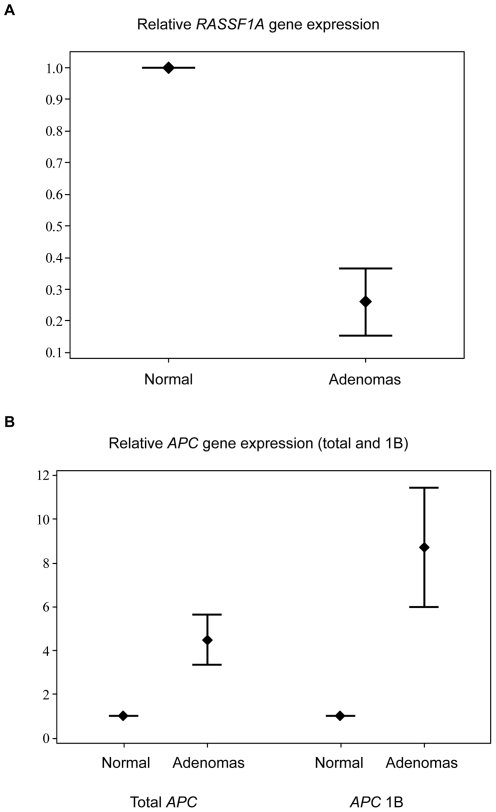
Interval plots demonstrating relative *RASSF1A* and *APC* gene expression in parathyroid tumours. 16 adenomas were compared to the normal parathyroid mean regarding (**A**) *RASSF1A* and (**B**) total *APC* and *APC* 1B gene expression. The endpoints of the intervals represent the 95% confidence interval for the mean, and the diamond corresponds to the mean value.

For *APC*, we assessed both total levels of *APC* mRNA and an *APC* 1B specific transcript regulated by the *APC* promoter 1B. No TaqMan Gene Expression Assay exists exclusively for the *APC* 1A transcript. For the total levels of *APC* mRNA, all 16 parathyroid adenomas available for qRT-PCR exhibited greater relative expression levels compared to the normal parathyroid mean, ranging from 1.3–9 (normal parathyroid mean  = 1) and a mean relative expression of 4.5. For the *APC* 1B transcript, all tumours displayed greater relative expression levels compared to the normal parathyroid mean ranging from 2–20 (normal parathyroid mean  = 1) and a mean relative expression of 8.7 (Figure 4). For *RASSF1A*, all 16 adenomas investigated have been shown to exhibit *RASSF1A* hypermethylation ranging from 37.2–71.2%. Using qRT-PCR, all tumours were found to exhibit lower relative *RASSF1A* mRNA expression levels compared to the normal parathyroid mean value, ranging from 0.05–0,8 (normal parathyroid mean  = 1). The mean relative expression was 0.26, suggesting that parathyroid adenomas display a noteworthy reduction in *RASSF1A* expression compared to normal parathyroid (Figure 4).

## Discussion

In this study we demonstrate frequent hypermethylation of the *APC* and *RASSF1A* tumour suppressor genes in the majority of parathyroid adenomas. *RASSF1A* promoter hypermethylation led to diminished *RASSF1A* mRNA levels in parathyroid adenomas as compared to normal parathyroid tissues, whereas *APC* gene expression is retained through augmented expression of *APC* 1B mRNA regulated by *APC* promoter 1B. In addition, based on *LINE-1* promoter methylation results parathyroid benign tumours were found to exhibit global methylation levels within range of the normal parathyroid tissues examined.

*APC* gene expression is regulated by at least two separate promoter regions, named 1A and 1B. Hypermethylation of promoter 1A has been readily demonstrated in various human tumours; however to our knowledge there is no or little evidence suggesting epigenetic inactivation of the *APC* promoter 1B through hypermethylation [33]. Hence, for this purpose we focused solely on the *APC* promoter 1A regarding Pyrosequencing analyses. In our tumour panel, hypermethylation of the *APC* promoter 1A was found in the majority of benign parathyroid tumours, as well as in one familial *HRPT2* related case. These results are supported by research in colorectal tumorigenesis, where promoter 1A methylation is regarded as an early event and is readily detected in small colorectal adenomas [33]. Furthermore, in this study all 16 adenomas analyzed by qRT-PCR displayed augmented *APC* 1B mRNA levels as compared to normal parathyroid tissues, strongly suggesting that the *APC* promoter 1B is not epigenetically silenced in these tumours.

Parafibromin, encoded by *HRPT2*, is a vital part of the hPAF complex involved in epigenetic modifications for facilitating gene transcription, and has also been shown to specifically regulate *c-Myc* oncogene expression through binding directly to the *c-Myc* promoter region [7]. Our results do not point towards a specific correlation between *APC* promoter methylation and *HRPT2* gene mutations, suggesting that parafibromin may not play an apparent role in the regulation of *APC* promoter 1A methylation. However, *APC* promoter 1A methylation displayed a weak but significant correlation to the tumour suppressor *MEN1* genotype, and all parathyroid tumours with *MEN1* mutations in our panel displayed *APC* hypermethylation ranging from 11.6–65.9% (Figure 2). Interestingly, the *MEN1* protein product menin has recently been implicated as an epigenetic modifier of Axin2, a component within the *Wnt* pathway as well as an APC-associated protein [8]. Our study vaguely implies that menin might regulate the levels of *APC* promoter methylation at the DNA level in parathyroid cells, since cases with *MEN1* mutations in general displayed higher levels of methylation than wild-type cases. Further molecular studies are needed to investigate the potential role of menin as a DNA regulator of *APC* promoter methylation status in addition to its known role as an Axin2 regulator through histone methylation.

The *RASSF1A* promoter was found to be hypermethylated in nearly all parathyroid adenomas examined and *RASSF1A* gene expression levels were considerably reduced in adenomas compared to normal parathyroid tissue. These data strongly support the involvement of this tumour suppressor gene in parathyroid tumorigenesis. In unrelated tumour tissues, *RASSF1A* promoter methylation has been shown to greatly diminish the levels of *RASSF1A* mRNA transcription, and subsequent usage of demethylating agents such as 5-Aza-CdR reconstitutes the *RASSF1A* transcription levels [34]. The role of *RASSF1A* as a cyclin D1 posttranslational modifier motivated us to correlate our methylation data to previous cyclin D1 expression results for the parathyroid cystic adenomas published elsewhere [35]. However, no statistical correlation between increased *RASSF1A* promoter methylation density and over-expressed cyclin D1 levels were seen (data not shown), suggesting that *RASSF1A* hypermethylation at least in part might contribute to excessive parathyroid tumour growth by other means than cyclin D1 accumulation, for example through its role as a regulator of apoptosis and mitotic arrest [19].

Regarding *APC*, we demonstrate three cases of normal parathyroid tissue (Normal PT1-3) as well as a case of secondary hyperparathyroidism with hyperplasia (case SHPT1) displaying virtually no CpG promoter methylation, suggesting that methylation of this locus might be conserved to true tumorous lesions of the parathyroids, such as adenomas and carcinomas. Moreover, studies have shown that normal adrenal as well as normal colonic mucosa in general shows *APC* promoter 1A methylation levels below 5%, as compared to the higher levels observed in the corresponding tumours [20], [32]. Also, the vast majority of cases of normal breast and lung tissue display an un-methylated *APC* promoter 1A as compared to the corresponding tumours [15], [36]; consequently these are examples which illustrate the tumour-specific phenotype for *APC* promoter 1A methylation in some tissue types.

For *RASSF1A*, the three normal parathyroid samples were endowed with virtually no methylation (2.0%, 3.6% and 3.0% respectively) whereas case SHPT1 was endowed with a methylation density of approximately 30%, suggesting that *RASSF1A* methylation also can be found in parathyroid tissue with an disproportionate growth pattern, albeit not a tumour per definition. Interestingly, cyclin D1 over-expression is also found in a fraction of cases with secondary hyperparathyroidism, suggesting that a subset of these lesions are propelled in parts by processes similar to those present in parathyroid adenomas and carcinomas [37]. Hence, the finding of *RASSF1A* hypermethylation in case SHPT1 could imply that this process could contribute to pathological growth in secondary hyperparathyroidism in addition to adenomas. In other non-neoplastic endocrine tissues such as the normal adrenal medulla and normal thyroid tissue, the *RASSF1A* promoter habitually exhibit low levels (<10%) of methylation densities with few exceptions [20]–[21]. In our study, normal thymus and leukocyte DNA was used as additional controls and displayed low levels of *RASSF1A* methylation, validating the specificity of our findings. Furthermore, a tumour case in our study displayed virtually no *RASSF1A* promoter methylation (CAD13, 8%), suggesting that parathyroid tissue not routinely exhibit high levels of methylation at this locus.

It is not known whether the hypermethylation of *APC* promoter 1A bears any pathological consequences to parathyroid growth, since APC protein expression has been previously demonstrated in parathyroid adenomas [16], [27]. However, a recent study has shown reduced *APC* promoter 1A mRNA levels in colorectal tumours with *APC* promoter 1A hypermethylation although retained APC protein expression through promoter 1B activity, and the authors advocate that even a slight decrease of *APC* gene transcription through the epigenetic silencing of promoter 1A could have an impact on Wnt signalling, although via unknown mechanisms [33]. Furthermore, another study regarding colorectal cancer found no correlations between *APC* methylation levels and APC protein expression [38]. In this study, we found notably augmented levels of total *APC* mRNA levels (from promoters 1A and 1B) as well as *APC* 1B mRNA in the adenomas investigated by qRT-PCR, of which 13 out of 16 samples displayed *APC* promoter 1A hypermethylation (Figure 4). These data imply that *APC* 1B mRNA is highly expressed in parathyroid adenomas, suggesting that the *APC* promoter 1B is not epigenetically inactivated in these cases. This presents a credible explanation to the fact that parathyroid adenomas display retained *APC* protein expression as demonstrated in previous publications although possibly endowed with *APC* promoter 1A hypermethylation [16], [27]. Furthermore, the finding of elevated levels of total *APC* mRNA is most likely a result of elevated levels of *APC* 1B mRNA expression, and does not argue directly against the theoretical, specific down-regulation of the *APC* 1A mRNA, the latter which is an observed phenomenon in colon cancer research [33]. In studies of unrelated tumour tissues, *APC* promoter 1A methylation is coupled to down-regulation of *APC* 1A mRNA, and treatment with demethylating agents results in re-expression of the specific transcript, demonstrating the sensible correlation between *APC* promoter methylation and *APC* mRNA transcript levels *in vivo* [39].

*LINE-1* bisulphite Pyrosequencing analysis has been shown to be an efficient substitute for global methylation assays [31], and our data thus suggests that parathyroid tumours display comparable methylation levels globally as normal parathyroid cells. This finding was unexpected, considering the current notion that human cancers generally exhibit global hypomethylation. In support of our findings, a current study recently demonstrated global hypermethylation in patients with myelodysplastic syndrome (MDS) as compared to normal controls using *LINE-1* Pyrosequencing [40]. The authors furthermore observed noteworthy clinical improvement among MDS patients when treated with hypomethylation-inducing agents, suggesting that the global hypermethylation was of clinical significance in this tumour type. Hence, these data suggest that not all tumours display relative hypomethylation globally compared to their corresponding normal tissues. In addition, we found that male cases displayed significantly higher levels of *LINE-1* methylation than female cases (Pearson's correlation −0.466, p<0.001), and cases with higher age at surgery displayed lower levels of *LINE-1* methylation than younger cases (Pearson's correlation 0.320, p = 0.018). These observations may suggest an influence of age or gender upon global levels of methylation in parathyroid tissues.

In summary, this study is the first to identify *APC* and *RASSF1A* promoter hypermethylation in the majority of parathyroid tumours investigated. The frequently observed hypermethylation of the *RASSF1A* promoter and downregulation of *RASSF1A* mRNA levels in parathyroid adenomas point toward an association between the inactivation of this tumour suppressor gene and parathyroid aberrant growth, whereas *APC* gene expression is retained in parathyroid adenomas due to increased transcription from *APC* promoter 1B. Moreover, *LINE-1* analyses indicate that parathyroid tumours do not exhibit global hypomethylation as compared to normal parathyroid tissues.

## Materials and Methods

### Ethics Statement

Fifty-five samples of parathyroid adenoma were collected with informed verbal consent. Informed verbal consent constitutes standard procedure at the Karolinska University Hospital, and the consent is subsequently documented in the patient's medical journal as according to Swedish Biobank law. The procedure has been specifically approved by the Karolinska University Hospital Ethics Committee and by the Karolinska Institute Research Ethics Committee, and these approvals include collection of normal parathyroid biopsies as well as normal tissues (including blood and thymus tissue) whenever motivated from patients undergoing surgery for primary and secondary hyperparathyroidism.

### Patients and Tissue Samples

Fifty-five samples of parathyroid adenoma were collected for the study (Tables 1 and 3, Table S1). Histopathological classifications were according to the guidelines of the World Health Organization (WHO) [41]. Thirty-four cases were parathyroid adenomas with cystic features (CAD1-34), which have previously been reported in detail including screening for *HRPT2* and *MEN1* mutations [3], [35]. Thirty-three of these cases were sporadic and one single case (CAD11) was derived from a 1q linked FIHP family. In addition one case (CAD25) was classified as an atypical adenoma. Two additional *HRPT2* related adenomas were derived from patients with germ-line *HRPT2* mutations (H1-2). Tumor H1 exhibited atypical histological findings that are sometimes seen in malignant parathyroid tumours, but was without definite criteria of malignancy in agreement with the diagnosis atypical adenoma or equivocal carcinomas as previously applied [42]. Nineteen cases were regular parathyroid adenomas (RAD1-19) that have been previously characterized for *MEN1* genotype [43]–[44]. Moreover, a case of parathyroid hyperplasia due to secondary hyperparathyroidism (SHPT1) was also included in the study. As non-tumorous normal controls, three samples of normal parathyroid tissue derived from surgical biopsies were included together with human leukocyte DNA from patient RAD2 as well as thymic tissue from patient H1.

### Bisulphite Treatment of DNA and Pyrosequencing Analyses

Sodium bisulphite modification of 500 ng DNA was carried out with the EZ DNA methylation kit (D5002, Zymo Research Corporation, CA, USA) following the manufacturer's protocol. The subsequent PCR and Pyrosequencing analysis to assess the level of methylation was carried out essentially as previously described for 10 CpG sites of the *APC* promoter 1A [20], 7 CpG sites of the *p16^INK4A^* promoter [45], 5 CpG sites of the *RASSF1A* promoter [46], 10 CpG sites of the *RAR-β* promoter [20] and 3 CpG sites of the *LINE-1* promoter [20]. Primer sequences are available at the PyroMark Assay Database (Biotage, Sweden) for all assays with the exception of *p16^INK4A^*, and were designed to hybridize with CpG-free regions to secure methylation-independent amplification. HotStar Taq polymerase was used to amplify 1 µl of bisulphite–treated DNA. PCR conditions were 95°C 15 min followed by 45 cycles of: 95°C 20 s, 50°C (*RAR-β* and *LINE-1*) or 55°C (*APC*, *RASSF1A*, *p16^INK4A^*) 20 s, 72°C 20 s, followed by 72°C 5 min and finally 4° C. All samples and blank controls for each reaction (PCR water control and additional non-PCR water control) were then analyzed using Pyrosequencing analysis and the PyroMark Q24 system (Biotage). Subsequent quantification of methylation density was done using the PyroMark Q24 software. Cases of bisulphite-treated normal leukocyte and thymic DNA were assessed as additional negative controls. Subsets of cases were re-analyzed with repeated PCR and Pyrosequencing reactions to test the validity of the initial Pyrosequencing findings for all promoter regions included, with similar end results (data not shown). Subsets of cases (including both tumour and normal parathyroid samples) were also repeatedly bisulphite treated and subsequently analyzed using PCR and Pyrosequencing, with analogous results (data not shown).

We chose to define hypermethylation as an average CpG residue methylation above 10% for *APC*, *RASSF1A*, *p16^INK4A^* and *RAR-beta* since the normal parathyroid mean as well as other normal tissues in this study were below this value.

### RNA Isolation, cDNA Synthesis and Quantitative RT-PCR (qRT-PCR)

Total RNA was available for 16 of the parathyroid adenomas and two of the normal parathyroid glands (normal PT1-2). All samples were extracted using the TRIzol Reagent (Invitrogen, CA, USA) and subsequently purified with the RNeasy kit (Qiagen, CA, USA). The RNA quality was verified using the NanoDrop ND-1000 (Thermo Scientific, DE, USA) for confirmation of acceptable 260/280 nm absorbance ratio as well as determining the sample concentrations. In addition, all RNA samples were analyzed in agarose gels where the integrity of the 28S and 18S bands was verified. Subsequent cDNA synthesis was performed using the High Capacity cDNA Reverse Transcription Kit (Applied Biosystems, CA, USA) following the company protocol. Subsequent assessment of *APC* and *RASSF1A* mRNA levels was carried out by quantitative RT-PCR (qRT-PCR) using the TaqMan Gene Expression Assay Technique and the 7900HT Fast Real-Time PCR System (Applied Biosystems, CA, USA). For *APC*, two assays were included; Hs00181051_m1 which covers total levels of *APC* mRNA (covering three transcript variants, one regulated from promoter 1A and two from 1B) and Hs01568282_m1 which covers the specific *APC* 1B transcript regulated by promoter 1B. There is no assay available exclusively for the analysis of the *APC* 1A transcript regulated by the *APC* promoter 1A using the TaqMan Gene Expression Assay technique. The *RPLP0* (ribosomal protein, large, P0), also known as *36B4*, has been used in previous publications by the authors and was selected as a housekeeping reference known to demonstrate little variation in parathyroid tissues (Hs99999902_m1) [35]. For the *APC* promoter 1A, 13/16 adenomas available for qRT-PCR displayed hypermethylation ranging from 10.3–64.1%. For *RASSF1A*, all 16 adenomas analyzed demonstrated *RASSF1A* promoter hypermethylation ranging from 37.2–71.2%. The two normal parathyroid tissues analyzed were devoid of hypermethylation covering both the *RASSF1A* and *APC* promoters.

A standard curve was established for relative quantification using cDNA from tumour case CAD20, and the qRT-PCR was carried out in 25 µl reactions with 12.5 µl TaqMan Gene Expression Master Mix, 8.5 µl RNase free H_2_O, 1 µl TaqMan Gene Expression Assay and 3 µl cDNA. The thermocycling conditions were 50°C for 2 min, 95°C for 10 min followed by 40 cycles of 95°C for 15 sec and 60°C for 1 min. Two non-template controls were assessed as negative controls for each experiment, and all experiments were carried out in double. After quantification relative to the standard curve, all samples were normalized to their corresponding *RPLP0* value and thereafter the normal parathyroid mean for each assay.

### Statistical Analyses

Possible correlations between methylation density in the different promoters assayed as well as between individual promoters and clinical/genetic characteristics were evaluated using the Minitab 15 statistical software (Minitab Inc, PA, USA). Correlations were appraised by Pearson's correlation and differences between groups were verified using Fisher's exact test. P-values <0.05 were considered as statistically significant.

## Supporting Information

Table S1 (0.03 MB XLS)
